# Supplementary material for: Distance-dependent seed‒seedling transition in the tree Castanopsis sclerophylla is altered by fragment size
Source: Commun Biol. 2019 Jul 26;2:277. doi: 10.1038/s42003-019-0528-x (PMC6659698; doi:10.1038/s42003-019-0528-x)
Supplement: Supplementary file 1 — Reporting Summary [file 42003_2019_528_MOESM1_ESM.pdf]

nature research

Corresponding author(s): Xiao-Yong Chen COMMSBIO-19-0121B

Last updated by author(s): Jun 19, 2019

Reporting Summary

Nature Research wishes to improve the reproducibility of the work that we publish. This form provides structure for consistency and transparency in reporting. For further information on Nature Research policies, see Authors & Referees and the Editorial Policy Checklist.

Statistics

For all statistical analyses, confirm that the following items are present in the figure legend, table legend, main text, or Methods section.

n/a	Confirmed	
☐	☒	The exact sample size (*n*) for each experimental group/condition, given as a discrete number and unit of measurement
☐	☒	A statement on whether measurements were taken from distinct samples or whether the same sample was measured repeatedly
☐	☒	The statistical test(s) used AND whether they are one- or two-sided<br>*Only common tests should be described solely by name; describe more complex techniques in the Methods section.*
☐	☒	A description of all covariates tested
☐	☒	A description of any assumptions or corrections, such as tests of normality and adjustment for multiple comparisons
☐	☒	A full description of the statistical parameters including central tendency (e.g. means) or other basic estimates (e.g. regression coefficient) AND variation (e.g. standard deviation) or associated estimates of uncertainty (e.g. confidence intervals)
☐	☒	For null hypothesis testing, the test statistic (e.g. *F*, *t*, *r*) with confidence intervals, effect sizes, degrees of freedom and *P* value noted<br>*Give P values as exact values whenever suitable.*
☒	☐	For Bayesian analysis, information on the choice of priors and Markov chain Monte Carlo settings
☐	☒	For hierarchical and complex designs, identification of the appropriate level for tests and full reporting of outcomes
☐	☒	Estimates of effect sizes (e.g. Cohen's *d*, Pearson's *r*), indicating how they were calculated

Our web collection on statistics for biologists contains articles on many of the points above.

Software and code

Policy information about availability of computer code

Data collection	No software was used, and all data were directly obtained from our experiments.
Data analysis	All analyses were carried out using the R packages lme4 version 1.1-12 and lmerTest version 3.0-1 in the programme R version 3.4.4, and all codes for analyses have been provided in the Methods in the manuscript.

For manuscripts utilizing custom algorithms or software that are central to the research but not yet described in published literature, software must be made available to editors/reviewers. We strongly encourage code deposition in a community repository (e.g. GitHub). See the Nature Research guidelines for submitting code & software for further information.

Data

Policy information about availability of data

All manuscripts must include a data availability statement. This statement should provide the following information, where applicable:

- Accession codes, unique identifiers, or web links for publicly available datasets
- A list of figures that have associated raw data
- A description of any restrictions on data availability

All data have been deposited in Dryad with the same title as the manuscript.

Field-specific reporting

Please select the one below that is the best fit for your research. If you are not sure, read the appropriate sections before making your selection.

☐ Life sciences ☐ Behavioural & social sciences ☒ Ecological, evolutionary & environmental sciences

Ecological, evolutionary & environmental sciences study design

All studies must disclose on these points even when the disclosure is negative.

Study description	We surveyed the seed–seedling transition process for four years using a total of 25,500 seeds of a local dominant tree species on islands with various sizes in a reservoir and on the nearby mainland, and found that the negative distance-dependence of seed-seedling transition was altered by habitat size. Specifically, we selected four types of habitats: (1) mainland: two large forest patches (> 300 ha); (2) large island: the only large island (875 ha); (3) medium islands: three medium-sized islands (13~ 51ha); and (4) small islands: four small islands (1.1-3.9 ha). Three experimental sites with a minimum interval of 2 km were established on the mainland and the large island, respectively, and one experimental site was set up on each of the selected medium and small islands. Therefore, at least three experimental sites were set in each fragment type. In each experimental site, we surveyed 500 seeds in each experimental year and recorded their conditions and distances to the nearest large conspecific tree and seed. Hence, the study design is hierarchical with seeds nested in experimental sites, which were nested in fragment types, and such design structure as well as different years were considered as the random effects in all corresponding analyses.<br>In addition, we also survey local rodent community and the seeds that were infested by pathogens and found that they were also affected by habitat size and contributed significantly to the formation of spatial seed-seedling transition patterns. Corresponding capture-recapture experiments were carried out in all experimental sites, and the hierarchical structure of data and different years were also considered in data analyses when setting random effects.
Research sample	Castanopsis sclerophylla (Fagaceae), a monoecious nut-bearing tree, is one of the dominant trees on most of the islands in the southeast of the reservoir. Early studies showed that rodents are the primary seed consumer and primary media of seed dispersal. Therefore, negative distance-dependence may function in regulating the population size of C. sclerophylla. This priori knowledge was the main reason to choose this local dominant species for the study. Additionally, C. sclerophylla is common in other Chinese subtropical evergreen broadleaved forests, and thus insights from this study may have general application in other subtropical evergreen forests.
Sampling strategy	In autumn of each experimental year, intact Castanopsis sclerophylla seeds were collected from at least 50 adult trees in all experimental sites and were fully mixed for the seed-seedling transition experiments. In each experimental site, 500 seeds were placed and monitored. The number of seeds per site is determined in our preliminary experiment in 2006.
Data collection	In the seed–seedling transition experiments, we monitored seed dispersal, over-winter survival and seedling emergence for all seeds used in the experiments. In seed dispersal stage, each day after the initial placement of seeds, we checked the number of seeds remaining and carefully searched the seeds dispersed away until all seeds were removed or eaten in each experimental site. All intact and destroyed seeds after dispersal were mapped, and their distances to the nearest conspecific tree (height > 1.5 m) (DTNCTs) were measured. In addition, we measured the distance from an intact seed to the nearest intact seed (DTNIS). In the following stages, the conditions (intact or not; or seedling established or not) of seeds were also recorded and their DTNCTs and DTNISs were measured.<br>In the rodent capture-recapture experiments, we identified and marked the rodents captured by rodent cages and released them for recapture, to estimate the rodent population density in each experimental site.<br>Data were measured and collected from the above experiments by Rong Wang, Yi-Su Shi, Yu-Xuan Zhang, Gao-Fu Xu, Qian Zhang, Bin Ai, and Yuan Miao.
Timing and spatial scale	During 2009 – 2012, we repeated (for four times) the seed–seedling transition experiment every year from autumn to the next spring in all experimental sites. Besides, during 2010-2013, the rodent capture-recapture experiment was repeated (for four times) in January. At the beginning of seed-seedling transition experiments (at seed dispersal stage), we checked the experimental sites and recorded data daily because most seeds were removed by rodents soon after being placed, and the interval between field survey became 10 days at over winter survival and seedling emergency stages since seeds/seedlings were not frequently attacked by rodents. Our experimental sites were located in the habitats (islands and the nearby mainland) with areas ranging from 1.1 ha to larger than 1000 ha, providing huge span for testing our habitat size-related hypothesis.
Data exclusions	No data were excluded from the analyses.
Reproducibility	Although the field environmental conditions changed year by year, we repeated our field experiments for four years and generally the results from the four-years experiments showed similar patterns of seed-seedling transition.
Randomization	To ensure sampling randomization, seeds used in the seed-seedling transition experiments were collected from at least at least 50 adult trees in all experimental sites and were fully mixed. Furthermore, we considered the hierarchical structure of data and different years as random effects in all analyses in case of the independence of data.
Blinding	Different groups of researchers and postgraduates conducted the experiments in each year, without informing the results obtained from the experiments in the previous year(s). Analyses were carried out using an open source statistical programme R, and therefore the algorithm was not specifically designed for our data.

Did the study involve field work? ☒ Yes ☐ No

Field work, collection and transport

Field conditions	The study area was mainly covered by subtropical evergreen broadleaved forests and was dominated by subtropical monsoon climate with the average temperature of 17 degrees Celsius and the mean precipitation of 1400 mm.

Location	Our study was carried out in the southeastern Thousand-Island Lake (N 29º30'-29º35', E 119º02'-119º09'), a typical land-bridge island system formed by Xin'an Jiang Dam in 1959, in Zhejiang Province, China. The elevation of experimental sites ranged from 105 m to 121 m, and all experimental sites were located on the islands and the nearby mainland with areas of continuous forests ranging from 1.1 ha to larger than 1000 ha.
Access and import/export	The forests in our study area were not included in nature reserves that required permits for entrance, and our study did not involve import/export of samples because all sampled seeds were used in the local field experiments.
Disturbance	The experiments in our study mainly included the field survey of the seed-seedling transition of a local dominant tree species and population density of rodents, and therefore we did not disturb the local biotic and abiotic environments. Moreover, the tags used to track the dispersed seeds were made of degradable plastics and are not likely to cause soil pollution.

Reporting for specific materials, systems and methods

We require information from authors about some types of materials, experimental systems and methods used in many studies. Here, indicate whether each material, system or method listed is relevant to your study. If you are not sure if a list item applies to your research, read the appropriate section before selecting a response.

Materials & experimental systems

n/a	Involved in the study
☒	☐ Antibodies
☒	☐ Eukaryotic cell lines
☒	☐ Palaeontology
☐	☒ Animals and other organisms
☒	☐ Human research participants
☒	☐ Clinical data

Methods

n/a	Involved in the study
☒	☐ ChIP-seq
☒	☐ Flow cytometry
☒	☐ MRI-based neuroimaging

Animals and other organisms

Policy information about studies involving animals; ARRIVE guidelines recommended for reporting animal research

Laboratory animals	The study did not involve laboratory animals.
Wild animals	In our study, we caught a total of 512 rodents belonging to two rat species (Niviventer fulvescens and Niviventer confucianus) in the field capture-recapture experiments using rodent cages. We did not kill any rodents, and the captive rodents were marked for population density estimation and then were immediately released.
Field-collected samples	The study did not involve samples collected from the field.
Ethics oversight	No ethical approval or guidance was required. Our experimental sites are not located in a conservation zone, and the studied rodents are not endangered species. In addition, we just surveyed local rodent density using rodent cages without collecting their tissues for any other experiments.

Note that full information on the approval of the study protocol must also be provided in the manuscript.
