# Supplementary material for: Distance-dependent seed‒seedling transition in the tree Castanopsis sclerophylla is altered by fragment size
Source: Commun Biol. 2019 Jul 26;2:277. doi: 10.1038/s42003-019-0528-x (PMC6659698; doi:10.1038/s42003-019-0528-x)
Supplement: Supplementary file 2 — Supplementary Information [file 42003_2019_528_MOESM2_ESM.pdf]

Supplementary information

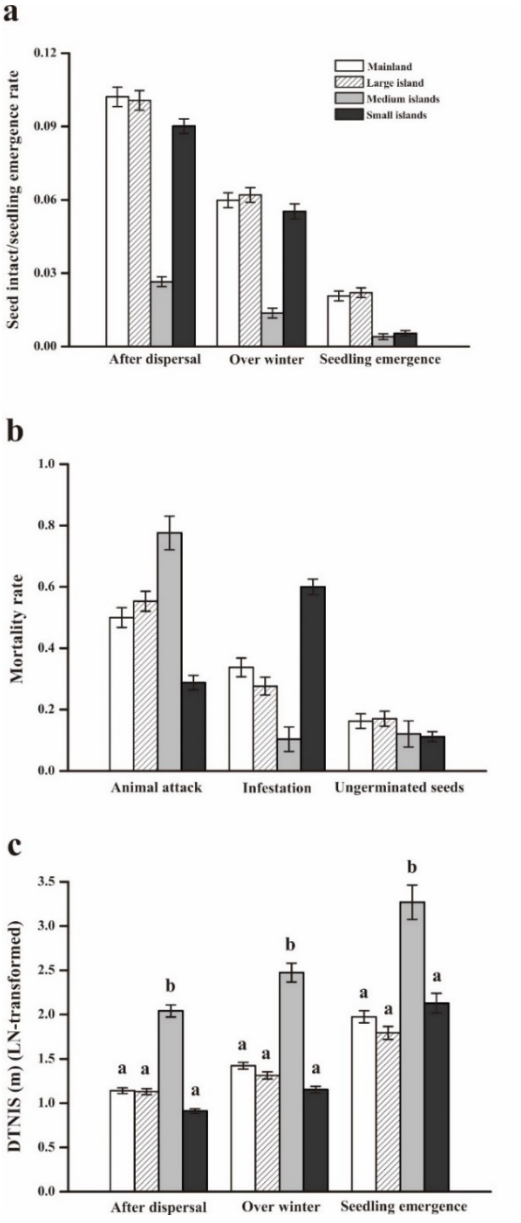

Supplementary Fig. 1 | Seed intact/seedling emergence rate (mean ± S.E.) at each seed–seedling transition stage (a), seed/seedling mortality rate caused by animal attack, infestation and un-germination at seedling emergence stage (b), and DTNIS at different stages (c) in all fragment types. (c) Different letters indicate significant differences based on the results from LMMs (see main Table 3).

Supplementary Table 1 | Comparisons of relative abundance, adult tree density (individuals/ha) and seed production (seeds/ha) of *C. sclerophylla* and DTNCTs of seed stations (m) among different fragment types, and rodent feeding preference (reflected by seed attack rate) among different tree species, using linear models (LMs), LMMs setting experimental sites nested in islands as random effects and generalized linear models (GLMs) assuming binomial distribution of residuals (with residual deviance and df of residuals). *P*-values of all tests were shown and Bonferroni correction was used to evaluate the significance for all pair-wise tests.

Response variable	Fixed effect	Models	LR tests		T/Z-tests		
			df	LR (*p*-value)	Pair-wise comparisons (mean ± S.E.)	df	t/z value (*p*-value)
Relative abundance (LN-transformed)	Fragment type	LMs	3	0.84 (0.507) NS	Mainland (0.711 ± 0.087) vs. large island (0.742 ± 0.036)	9	-0.35 (0.737) NS
					Mainland vs. medium islands (0.695 ± 0.081)	9	0.14 (0.893) NS
					Mainland vs. small islands (0.602 ± 0.065)	9	1.11 (0.296) NS
					Large island vs. medium islands	9	0.48 (0.640) NS
					Large island vs. small islands	9	1.48 (0.173) NS
					Medium islands vs. small islands	9	0.96 (0.362) NS
Adult tree density	Fragment type	LMs	3	0.77 (0.539) NS	Mainland (51.1 ± 6.0) vs. large island (64.1 ± 8.3)	9	-1.21 (0.258) NS
					Mainland vs. medium islands (54.5 ± 7.8)	9	-0.32 (0.758) NS
					Mainland vs. small islands (49.7 ± 6.9)	9	0.14 (0.895) NS
					Large island vs. medium islands	9	0.89 (0.397) NS
					Large island vs. small islands	9	1.43 (0.188) NS

					Medium islands vs. small islands	9	0.48 (0.645) ^{NS}
Seed production (LN-transformed)	Fragment type	LMs	3	0.28 (0.837) ^{NS}	Mainland (1986.4 ± 1056.7) vs. large island (2083.1 ± 332.4)	9	-0.47 (0.647) ^{NS}
					Mainland vs. medium islands (1637.4 ± 436.2)	9	-0.06 (0.955) ^{NS}
					Mainland vs. small islands (1688.7 ± 776.7)	9	0.41 (0.692) ^{NS}
					Large island vs. medium islands	9	0.42 (0.687) ^{NS}
					Large island vs. small islands	9	0.92 (0.384) ^{NS}
					Medium islands vs. small islands	9	0.47 (0.649) ^{NS}
DTNCTs of seed stations (LN-transformed)	Fragment type	LMMs	3	0.61 (0.895) ^{NS}	Mainland (4.56 ± 0.20) vs. large island (4.34 ± 0.19)	510	0.18 (0.855) ^{NS}
					Mainland vs. medium islands (4.47 ± 0.21)	510	0.19 (0.847) ^{NS}
					Mainland vs. small islands (4.78 ± 0.23)	510	0.73 (0.468) ^{NS}
					Large island vs. medium islands	510	0.01 (0.992) ^{NS}
					Large island vs. small islands	510	0.53 (0.595) ^{NS}
					Medium islands vs. small islands	510	0.52 (0.602) ^{NS}
Seed attack rate	Fagaceae species	GLMs (521, 597)	2	521.21 (<0.001) ***	*C. sclerophylla* (0.970 ± 0.012) vs. *L. glaber* (0.235 ± 0.030)	--	10.42 (<0.001) ***
					C. sclerophylla vs. *Q. glandulifera* (0.315 ± 0.033)	--	9.63 (<0.001) ***
					L. glaber vs. *Q. glandulifera*	--	-1.79 (0.074) ^{NS}

LR tests: likelihood ratio tests; ^{NS}: not significant; ***: p<0.001.

Supplementary Table 2 | Interaction between fragment size and DTNCT on the seed attack rate/the seed/seedling mortality rate at each seed–seedling transition stage, using GLMMs assuming binomial distribution of residuals.

Response variable	Model parameters		LR tests		Fixed effect	Z-tests	
	Residual deviance	df of residuals	df	LR (*p*-value)		Coefficient (mean ± S.E.)	z value (*p*-value)
Seed attack rate after dispersal	9719.4	10252	1	21.07 (<0.001) ***	Fragment size (LN-transformed)	0.13 ± 0.09	1.36 (0.174) ^{NS}
					DTNCT (LN-transformed)	0.02 ± 0.08	0.25 (0.800) ^{NS}
					Fragment size × DTNCT (Both LN-transformed)	-0.07 ± 0.01	-4.61 (<0.001) ***
Seed attack rate over winter	2623.4	2043	1	10.82 (0.001) **	Fragment size (LN-transformed)	0.11 ± 0.05	2.24 (0.025) *
					DTNCT (LN-transformed)	-0.39 ± 0.13	-3.00 (0.003) **
					Fragment size × DTNCT (Both LN-transformed)	-0.09 ± 0.03	-3.29 (<0.001) ***
Seed/seedling mortality rate	1244.6	1219	1	11.68 (<0.001) ***	Fragment size (LN-transformed)	0.03 ± 0.09	0.37 (0.714) ^{NS}
					DTNCT (LN-transformed)	0.13 ± 0.28	0.48 (0.631) ^{NS}
					Fragment size × DTNCT (Both LN-transformed)	-0.18 ± 0.05	-3.48 (<0.001) ***

^{NS}: not significant; *: p<0.05; **: p<0.01; ***: p<0.001.
